# Mononuclear ruthenium polypyridine complexes that catalyze water oxidation

**DOI:** 10.1039/c6sc02766k

**Published:** 2016-08-05

**Authors:** Lianpeng Tong, Randolph P. Thummel

**Affiliations:** a Department of Chemistry , University of Houston , 112 Fleming Building , Houston , Texas 77204-5003 , USA . Email: lianpeng.tong@fau.de ; Email: thummel@uh.edu

## Abstract

Over the past decade, significant advances have been made in the development of molecular water oxidation catalysts (WOCs) in the context of developing a system that would accomplish artificial photosynthesis. Mononuclear ruthenium complexes with polypyridine ligands have drawn considerable attention in this regard, due to their high catalytic activity and relatively simple structure. In this perspective review, we will discuss mononuclear Ru polypyridine WOCs by organizing them into four groups according to their ligand environments. Each group will be discussed with regard to three fundamental questions: first, how does the catalyst initiate O–O bond formation? Second, which step in the catalytic cycle is rate-determining? Third, how efficient is the catalyst according to the specific descriptors such as turnover frequency? All discussion is based on the high-valent ruthenium intermediates that are proposed in the catalytic cycle according to experimental observation and theoretical simulation. Two fundamental mechanisms are set forth. An acid–base mechanism that involves the attack of a water molecule on the oxo of a high valent Ru=O species to form the O–O bond. Subsequent steps lead to dissociation of O_2_ and rehydration of the metal center. A second mechanism involves the formation of a Ru–O˙ radical species, two of which then couple to form a Ru–O–O–Ru species that can release O_2_ afterwards. The acid–base mechanism appears to be more common and mechanistic differences could result from variation directly related to polypyridine ligand structures. Understanding how electronic, steric, and conformational properties can effect catalyst performance will lead to the rational design of more effective WOCs with not only ruthenium but also other transition metals.

## Abstract 2

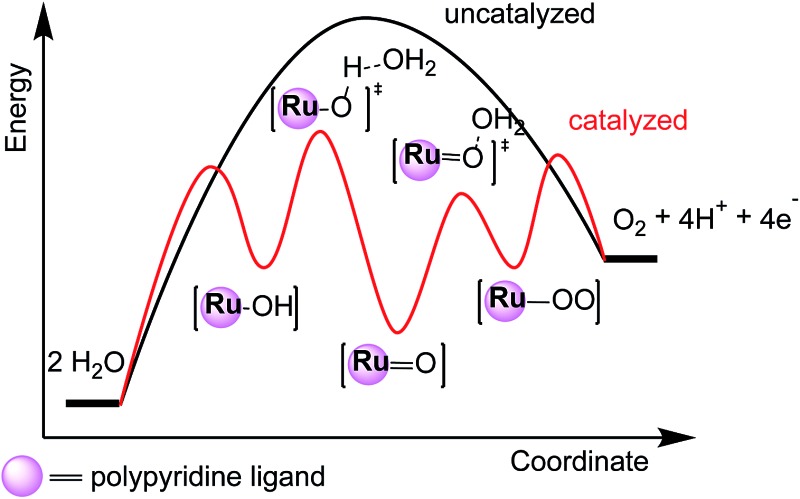
Representative mononuclear Ru polypyridine water oxidation catalysts were discussed by organizing them into four groups according to their ligand environments so as to elucidate the correlation between activity, mechanism, and ligand structure.

## 1. Introduction

The successful utilization of solar energy as an alternative to fossil fuels relies on the viable conversion of solar energy into ‘solar fuels’ that can be stored and distributed in a manner similar to fossil fuels.^1^ One approach to achieving this conversion is envisioned as an artificial photosynthesis (AP) system that mimics the function of the naturally-occurring photosynthetic system.^2^ The AP system includes two half reactions. From an electrochemical point of view, these reactions are the anodic water oxidation reaction (eqn (1)) and the cathodic solar fuel generation, such as proton to hydrogen or CO_2_ to methanol reduction. When the overall endothermic redox process is driven by sunlight, solar energy is converted into chemical energy in the form of chemical bonds and dioxygen is liberated concurrently. Exothermic oxidation of the solar fuel by dioxygen releases the energy and closes the energy cycle in a carbon-neutral way.

1 2H_2_O → O_2_ + 4H^+^ + 4e^–^, *E*
^0^ = 1.23 V

**Fig. 1:**
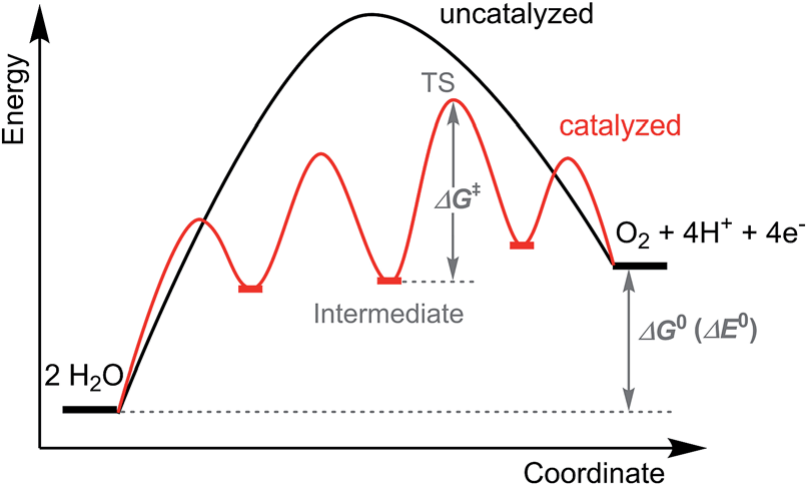
Schematic energy profiles for the uncatalyzed and catalyzed water to dioxygen reaction. The highest energy barrier (Δ*G* ^‡^) in the catalyzed path is marked. The transition state is denoted as TS.

The water oxidation reaction (eqn (1)) is ideally suited to either natural or artificial photosynthesis because water and dioxygen are the most abundant electron donor (reductant) and acceptor (oxidant) in the world. This oxidation reaction is energy demanding with a standard redox potential of 1.23 V (all redox potentials presented in this paper are *versus* standard hydrogen electrode, SHE, unless noted otherwise). In nature, water oxidation is catalyzed by the oxygen evolving complex (OEC) of Photosystem II (PS II).^3,4^ In an artificial system, a water oxidation catalyst (WOC) would be required to lower the energy barrier (Δ*G* ^‡^) of activation for this process. This situation can be illustrated by comparing the schematic energy profiles of catalyzed and uncatalyzed water oxidation pathways (Fig. 1). Water oxidation is a complex reaction that involves the removal of four electrons and four protons as well as the formation of the O=O bond. Multiple intermediates are likely to be involved in the catalytic pathway. An ideal WOC should avoid high-energy (‘too active’) and low-energy (‘too stable’) intermediates that are likely to require large energy barriers of activation. Thus, the rational design of a WOC becomes a task of manipulating critical intermediates throughout the catalytic cycle. This detailed description, in turn, relies on elucidating the critical intermediates and understanding the influence of structural factors upon their relative energies. In this regard, the study of molecular ruthenium WOCs during the past decade can provide some clues and inspiration. This review will be restricted to homogeneous Ru-based WOCs whose molecular structures are well defined.

## 2. Basic considerations

### 2.1 Polypyridine ligand platform

**Scheme 1:**
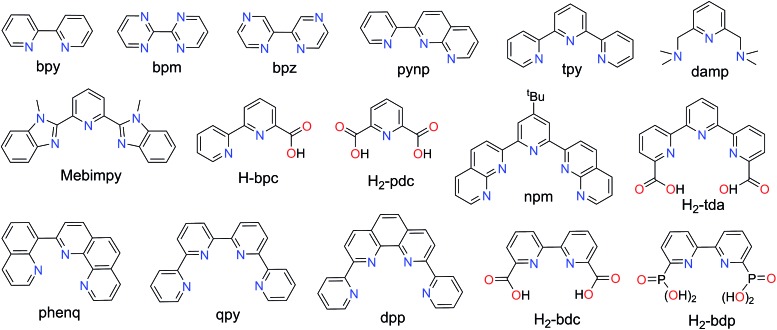
Selected polypyridine ligands discussed in this review.

Most molecular ruthenium water oxidation catalysts reported so far are based on polypyridine ligands that include the polypyridine backbone and non-pyridine donors such as imidazole or carboxylate (Scheme 1). These ligands were systematically designed and synthesized with careful concern given to their denticity, rigidity, and conjugation as well as the positioning of substituent groups having different steric and electronic effects. In this manner the influence of ligand features upon catalytic activity can be compared and illustrated. The suitability of polypyridine ligands is not a coincidence as they meet two basic requirements for catalytic water oxidation. Firstly, the pyridine ring is capable of tolerating harsh oxidation conditions and, secondly, the pyridine ring is stable towards hydrolysis.

When coordinated with Ru(ii), the major role of pyridine is to provide its lone pair of electrons as a σ-donor to the metal center. The pyridine–Ru coordination bond is quite effective and leads to large ligand field stabilization energy. As a result, Ru complexes with a pyridine coordinating environment prefer a low-spin electronic configuration. Polypyridines chelate with Ru through multidentate sites resulting in the formation of chelate rings. This multi-binding tethers the ligand and Ru firmly enough to resist ligand displacement by water under acidic or alkaline conditions. The polypyridine ligands are generally believed to be redox insensitive when the Ru(ii) complexes are oxidized to higher valences.

### 2.2 Proton-coupled electron transfer (PCET) and high valent ruthenium species

**Scheme 2:**
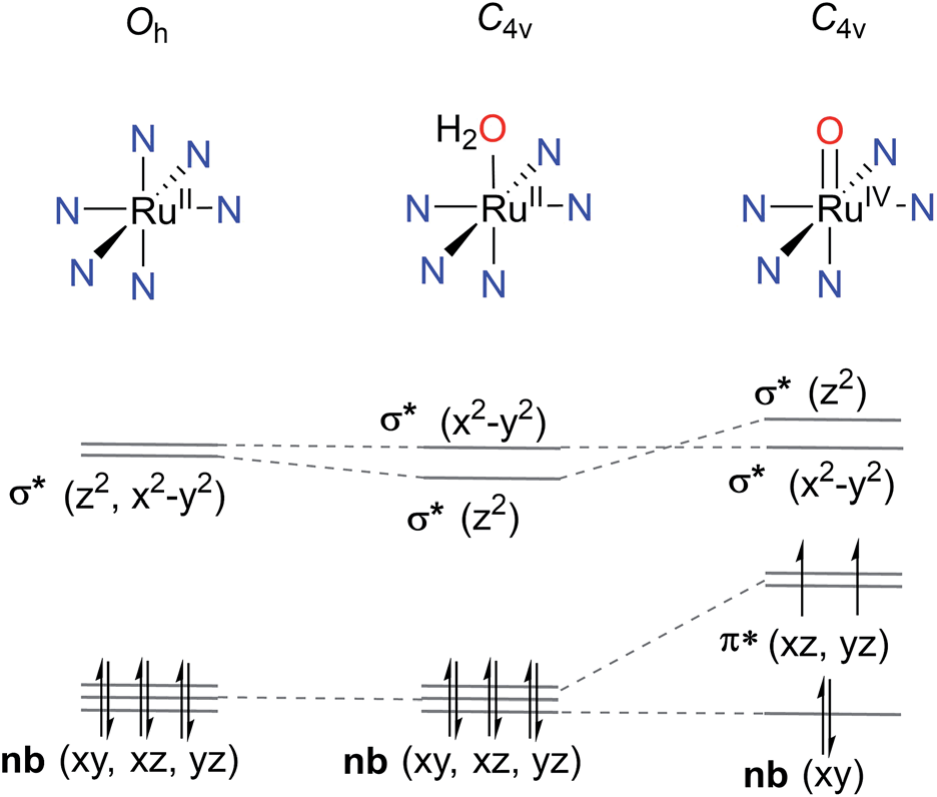
Schematic frontier molecular orbital diagrams for ruthenium complexes with a tetragonal ligand field.

The frontier molecular orbital diagram of an octahedral Ru(ii) complex with six identical pyridine ligands is shown in Scheme 2. It can be used as a simplified model to analyze related ruthenium polypyridine systems. Removal of one electron from the highest occupied molecular orbital (HOMO) of such a complex demands considerable energy. Oxidation of [Ru(bpy)_3_]^2+^ (bpy = 2,2′-bipyridine), for example, occurs at *E* ^1/2^(Ru^III^/Ru^II^) = 1.26 V in water.^5^ Changing one of the pyridine ligands for a water will stabilize the lowest unoccupied molecular orbital (LUMO) to some extent because the aqua ligand is a weaker σ-donor than pyridine. Nevertheless, this change does not significantly influence the HOMO orbital of the Ru(ii) complex (Scheme 2). As suggested in a study of [Ru^II^(bpy)_2_(py)(OH_2_)]^2+^ (py = pyridine), the standard redox potential of [Ru^III^(bpy)_2_(py) (OH_2_)]^3+^/[Ru^II^(bpy)_2_(py)(OH_2_)]^2+^ is 1.04 V, lower than but close to that of [Ru(bpy)_3_]^2+^.^6,7^ This complex in its trivalent state, however, is a much stronger Brønsted acid than in the divalent state. The p*K*_a_ of [Ru^III^(bpy)_2_(py)(OH_2_)]^3+^ and [Ru^II^(bpy)_2_(py)(OH_2_)]^2+^ are 0.85 and 10.20, respectively.^8,9^ In the pH range from 0.85 to 10.20, the PCET redox process of [Ru^III^(bpy)_2_(py)(OH)]^2+^/[Ru^II^(bpy)_2_(py)(OH_2_)]^2+^ becomes dominant with the redox potential depending on pH, according to the Nernst equation.^10^ As a result, the Ru(iii) state is thermodynamically easier to access at relatively higher pH (>0.85), for instance, 0.68 V at pH = 7.0.

Further oxidation of [Ru^III^(bpy)_2_(py)(OH)]^2+^ leads to [Ru^IV^(bpy)_2_(py)O]^2+^ (p*K*_a_ < –6.0).^11^ Because both the Ru^IV^–O/Ru^III^–OH and Ru^III^–OH/Ru^II^–OH_2_ redox events occur as a one-proton coupled one-electron transfers, their redox potentials change in parallel depending on pH (0.85–10.20). Notably, the potential gap is only 0.11 V between these two redox couples. By comparison, the potential difference between Ru^IV^/Ru^III^ and Ru^III^/Ru^II^ couples of *cis*-[Ru^II^(bpy)_2_Cl_2_]^2+^ is 1.66 V in MeCN.^12^ There are two major factors contributing to the dramatically narrow potential gap for aqua ruthenium complexes such as [Ru^II^(bpy)_2_(py)(OH_2_)]^2+^. One is the involvement of PCET that avoids charge buildup.^13^ The other is the interaction between the ruthenium d orbitals (d_*xz*_, d_*yz*_) and oxo p orbitals (p_*x*_, p_*y*_), which destabilize the HOMO by combining to form the dπ–pπ bonding and antibonding molecular orbitals.^14^ The π-bonding orbitals (not shown in Scheme 2) are centered on the oxo and are lower in energy than the nonbonding (*xy*) orbital, while the π* orbitals (HOMO, Scheme 2) are centered at the metal and are higher in energy. In this d^4^ electronic scenario of Ru^IV^ =O, the two lone pairs (p_*x*_ and p_*y*_) of oxygen are partially delocalized on the ruthenium center after the interaction. Meanwhile, the d_*xz*_ and d_*yz*_ orbitals, which are non-bonding before the interaction, partially delocalize onto the oxo ligand, leading to some electron transfer from ruthenium to oxygen.

For ease of reading, formal oxidation states of a ruthenium center are marked in this paper by assuming that all electron transfer processes of complexes are metal-based. This is in accordance with the conventional view regarding transition metal complexes. It should be noted that spin density in principle distributes over the whole molecule of a metal complex and electron transfer may occur primarily at the ligand such as oxo ligand (see below).

### 2.3 The O–O bond formation

How the O–O bond is formed is a vital aspect of the mechanism of catalytic water oxidation. Due to the requirement for multiple electron transfers in the water to dioxygen oxidation, Ru intermediates with various valence states have to be involved in the catalytic cycle. High valence (Ru^IV^ or Ru^V^) ruthenium oxo species are often postulated as critical intermediates that trigger O=O bond formation.

**Scheme 3:**
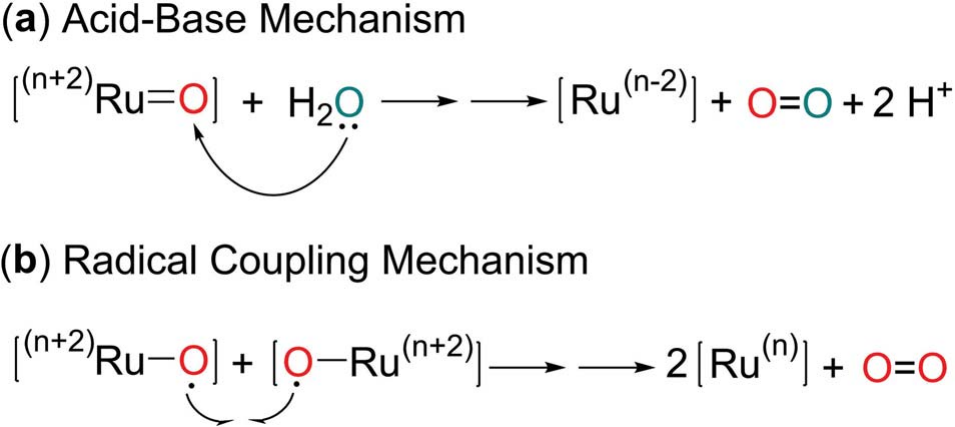
General pathways of O–O bond formation mediated by Ru-oxo intermediates.

There are two general mechanisms for O–O bond formation mediated by Ru-oxo species, according to the origin of the oxygen atoms in the generated dioxygen. In the acid–base mechanism (Scheme 3a), water or hydroxide as a Lewis base attacks the terminal oxo group as a Lewis acid. In the radical coupling mechanism (Scheme 3b), two radical-like Ru-oxo species approach and couple with each other. Therefore the favored pathway partly depends on the dominant resonance contributor between Ru^*N*^ =O and Ru^(*N*–1)^–O˙, such as Ru^V^ =O and Ru^IV^–O˙, under the reaction conditions. Kinetically, the two pathways may compete with each other. The essential high-valent Ru-oxo species are usually unstable and have only transient lifetimes in the reaction medium. This short lifetime makes direct characterization and observation of these species difficult. In principle, the two pathways for O–O bond formation can be distinguished experimentally by an ^18^O-labeled Ru-oxo or water substrate. Moreover, these two O–O bond formation steps show different kinetic orders in the ruthenium-containing intermediates.

### 2.4 Catalytic activity

The activity of molecular Ru WOCs can be described by overpotential (*η*) and turnover frequency (TOF). The former refers to the difference between the thermodynamic water oxidation potential and the catalytic potential (*E*_cat_) where an appreciable catalytic current is achieved.^15^ The latter is straightforwardly defined as the number of catalytic cycles mediated by each catalyst molecule per unit time. These two descriptors are not independent parameters but are linked to each other, because both are related to the activation energy of the rate-determining step (Fig. 1) in the catalytic cycle. Savéant and coworkers have developed electrochemical models to quantitatively characterize and analyze the TOF-η relationship for a molecular catalyst.^16–18^

The definition of *E*_cat_, however, is somewhat subjective and this parameter has been determined by cyclic voltammetry according to several different criteria. The potential at the onset, the maximum, or half of the maximum of catalytic current have all been designated as *E*_cat_. The different methods for estimation of *E*_cat_ lead to significant uncertainty concerning this parameter. Hence, caution should be taken in the direct comparison of catalytic potentials.

The TOF of Ru WOCs can also be evaluated by driving the catalyst with a sacrificial oxidant in bulk solution. The reduction potential of the oxidant should be positive enough not only to oxidize water thermodynamically but also to enable access to the highest valent intermediate present in the catalytic pathway. A number of sacrificial oxidants have been employed in catalytic water oxidation studies.^19^ Among them, ceric ammonium nitrate ([(NH_4_)_2_Ce^IV^](NO_3_)_6_, CAN) and [Ru(bpy)_3_]^3+^ are most often used under acidic and neutral conditions, respectively.^5,20,21^ Both are one-electron oxidants without O-transfer capability. This ensures that water is the only source of oxygen for O_2_ evolution. Because [Ru(bpy)_3_]^3+^ easily decomposes, even in the solid state, it is usually generated *in situ* through the exposure of [Ru(bpy)_3_]^2+^ to sodium peroxydisulfate and light.^22^ Alternatively, high purity CAN is commercially available and can be stored for long periods of time by avoiding moisture. Thus the preparation of a CAN solution with a given concentration is convenient and such a solution under acidic conditions (pH = 1.0) is commonly used in mechanistic studies of WOCs.

A high TOF at low overpotential is always desired for an efficient catalyst. The OEC of PSII is able to achieve a maximum TOF of about 500 s^–1^ under natural conditions and is often used as a benchmark for this process.^23^ For one mononuclear Ru WOC, a striking TOF of 50 000 s^–1^ was recently reported under electrocatalytic conditions.^24^

A Ru WOC may decompose and lose its activity during catalysis. Therefore, turnover number (TON) is also used to assess the catalytic behavior of WOCs. The TON can be defined as the number of oxygen molecules generated per molecule of catalyst before becoming inactivated. The value of the TON is related to both the efficiency and stability of the catalyst. It should be noted that the determination of TOF and TON is influenced by methodology and experimental conditions. In electrolysis, for example, the reaction rate may be limited by the diffusion of a substrate to the electrode surface, whereas a reaction in bulk solution is governed by the law of mass action. Therefore, how such descriptors of activity are determined should be provided when the catalytic behavior of different WOCs is compared.

## 3. Ruthenium polypyridine WOCs and their catalytic pathways

### 3.1 Blue dimer

**Fig. 2:**
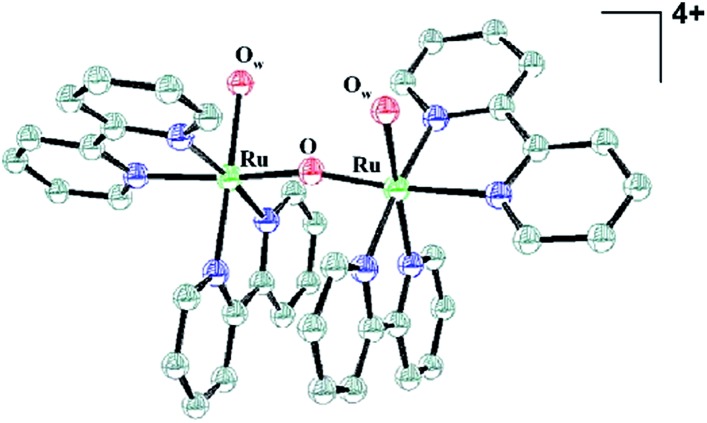
Thermal ellipsoid plot representation of the ruthenium blue dimer cation, *cis*,*cis*-[(bpy)_2_(H_2_O)Ru^III^ORu^III^(OH_2_)(bpy)_2_]^4+^. Reproduced with permission from ref. 30.

The so-called “blue dimer” (Fig. 2) was initially prepared and investigated by Meyer and coworkers during the early 1980's.^25–27^ It is the first ruthenium complex that was shown to be capable of catalyzing water oxidation. Under pH = 1 conditions, the blue dimer was oxidized from Ru^III^–O–Ru^III^ to Ru^V^–O–Ru^V^ at a potential >1.5 V *via* successive proton-coupled one-, and three-electron transfer processes (through the Ru^III^–O–Ru^IV^ state).^26^ The resulting [(O)Ru^V^(μ-O)Ru^V^(O)]^4+^ intermediate was believed to trigger the O_2_ evolution step. ^18^O-Labeling studies suggest a complicated mechanism that includes intra-, and inter-molecular coupling and acid–base types of interaction.^28,29^ A kinetic study using CAN illustrates the nucleophilic attack of water on the Ru^V^ =O center and the formation of a peroxo intermediate as the major catalytic pathway.^11,30^ This pathway is also supported by DFT calculations.^31^ Research involving the blue dimer has inspired the development of both dinuclear and mononuclear ruthenium WOCs using a variety of polypyridine ligands. The discussion of dinuclear Ru WOCs is outside of the scope of this review and we direct interested readers to related references.^32–37^

### 3.2 Mononuclear Ru polypyridine WOCs and their catalytic activity

**Scheme 4:**
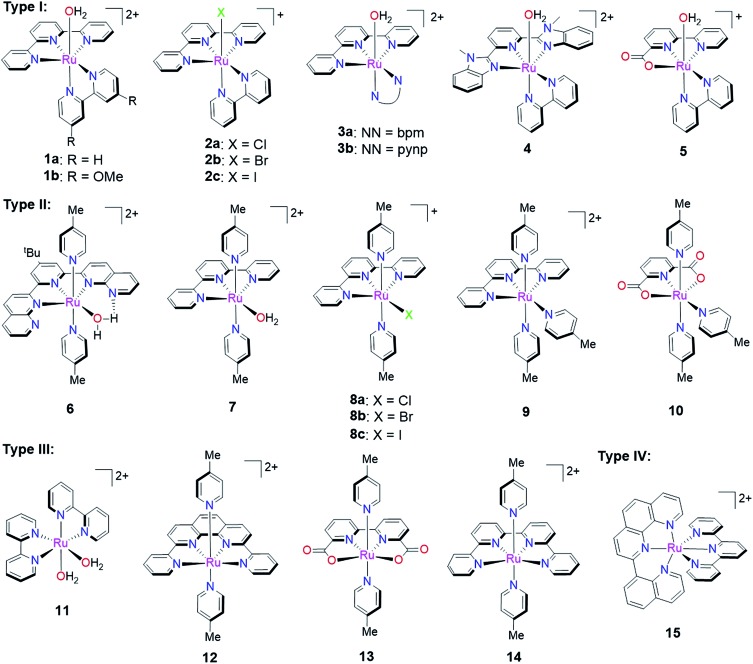
Selected mononuclear ruthenium WOCs having various polypyridine ligands.

In the past decade, a growing number of mononuclear Ru polypyridine complexes have been reported to catalyze the water oxidation reaction. Compared with multinuclear Ru WOCs, the mononuclear complexes have simpler structures, better-defined spectroscopic properties, and lend themselves more readily to functional group modification. Such mononuclear Ru WOCs thus provided an excellent opportunity for researchers to gain insight into catalytic pathways from both an experimental and theoretical point of view. It is difficult, however, to establish a straightforward correlation between the activity and specific features of these catalysts, because any given structural or electronic feature may simultaneously influence multiple steps in the catalytic pathway. In order to discuss these WOCs in a systematic manner, we will classify mononuclear ruthenium polypyridine WOCs into four groups according to their ancillary polypyridine scaffolds: (i) [Ru(LLL)(LL)X], (ii) [Ru(LLL)(L)_2_X], (iii) [Ru(LLLL)(L)_2_], and (iv) [Ru(LLL)_2_] types, where L, LL, LLL, and LLLL represent mono-, di-, tri-, and tetra-dentate N/O-polypyridine ligands, respectively, and X represents an aqua or halogen ligand. Instead of listing all reported Ru WOCs, we chose several representative examples from each group shown in Scheme 4 and concentrated on the differences in their catalytic behavior. For each catalyst, we will concern ourselves with the following three questions: (1) how does the catalyst initiate O–O bond formation? (2) Which is the rate-determining step in the catalytic cycle? (3) How does the ligand environment influence the catalytic activity according to the specific descriptors given in Section 2.4? The differences in catalytic behavior between complexes within one group mainly result from the different individual ligands and their various substituents. Nevertheless, complexes from different groups may have the same kind of ligand donors. For example, five N(pyridine)- and one aqua-ligands for both **1a** and **6**. Thus, their distinctive catalytic behavior derives from how these ligands are organized and ligated in space.

### 3.3 Type I: [Ru(LLL)(LL)X]^*n*+^ WOCs

**Scheme 5:**
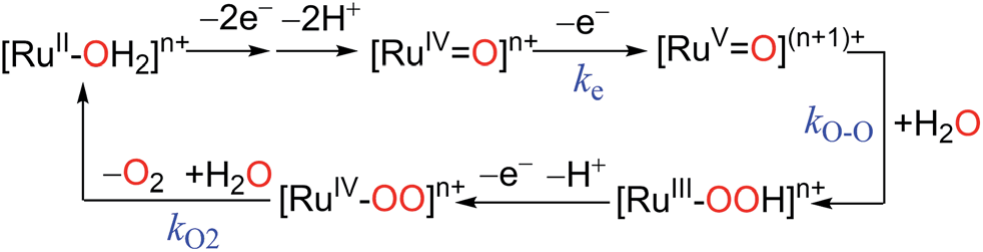
Generalized mechanism for water oxidation by Type I Ru catalysts in pH = 1.0 aqueous medium.

[Ru(LLL)(LL)X] type complexes constitute a major family of competent mononuclear WOCs. Mechanistic investigation reveals a general catalytic cycle under pH 1.0 conditions for this type of WOC, as depicted in Scheme 5.^38–40^ This catalytic cycle is consistent with the ‘acid–base’ mechanism. The reaction pathway begins with the oxidation of the Ru^II^ complex to its [Ru^IV^ =O] state *via* multiple PCET steps. A subsequent ET process (rate constant = *k*_e_) generates the high-valent [Ru^V^ =O] species. Nucleophilic water attack on [Ru^V^ =O] leads to the requisite O–O bond formation (rate constant = *k*_O–O_) and yields the hydroperoxide [Ru^III^–OOH], which undergoes another PECT step and generates the [Ru^IV^–OO] intermediate. At this stage, the dioxygen can readily dissociate from the metal center (rate constant = *k*_O_2__) and the original Ru^II^ complex is regenerated after water association. A competitive pathway involves a further oxidation of [Ru^IV^–OO] to [Ru^V^–OO] (not shown in Scheme 5), which is then reduced to the Ru^III^ state concomitant with O_2_ release.^40,41^ Despite the common catalytic path shared by Type I WOCs, the diversity of their ligand environments influences the kinetics and thermodynamics of critical steps in the cycle.

**Fig. 3:**
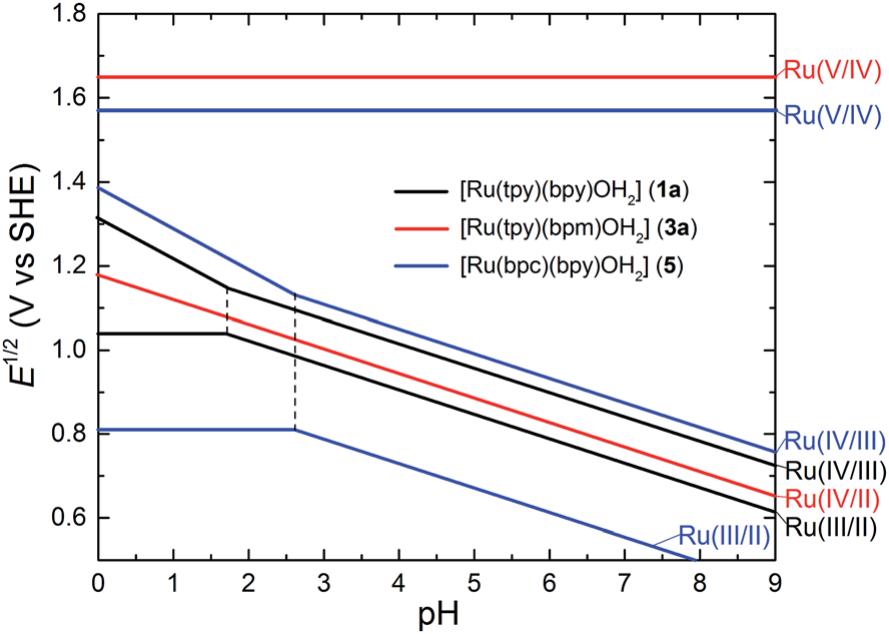
Pourbaix diagrams for [Ru(tpy)(bpy)OH_2_]^2+^ (**1a**), [Ru(tpy)(bpm)OH_2_]^2+^ (**3a**) and [Ru(bpc)(bpy)OH_2_]^2+^ (**5**); solid lines indicate trends of redox potentials depending on pH; dotted lines indicate p*K*_a_ of [Ru^III^–OH_2_] species. The diagram was drawn according to reported experimental data in refs. 38, 42 and 43.

[Ru^II^(tpy)(bpy)OH_2_]^2+^ (**1a**, tpy = 2,2′;6′,2′′-terpyridine) was studied extensively in the early 1980s'.^43^ The Pourbaix diagrams for **1** (black solid line in Fig. 3) shows either a [Ru^II^–OH_2_]^2+^ → [Ru^III^–OH]^2+^ → [Ru^IV^ =O]^2+^ or a [Ru^II^–OH_2_]^2+^ → [Ru^III^–OH_2_]^3+^ → [Ru^IV^ =O]^2+^ redox sequence, depending on whether the pH of the medium is greater or less than the p*K*_a1_ (1.7) of the [Ru^III^–OH_2_]^3+^ intermediate. At pH = 1.0 where [Ru^III^–OH_2_]^3+^ is not deprotonated, redox potentials of 1.04 and 1.23 V are determined for the Ru^III/II^ and Ru^IV/III^ couples, respectively. There is still some controversy about the existence of the Ru^V^ state of **1**,^40,45^ and no absorbance feature for a Ru^V^ species was observed by mixing 1 equiv. of CAN with the [Ru^IV^ =O]^2+^ form of **1a**.^44^ Nevertheless, the catalytic activity of **1a** toward water oxidation has been independently confirmed by several groups.^21,40,45–48^ A TOF of 6.1 × 10^–5^ s^–1^ was observed for **1a** in the presence of excess CAN (200 equiv.) under pH 1.0 conditions.^44^ The rate of CAN consumption depended on the concentration of **1a** but not the concentration of CAN, inferring either *k*_O–O_ or *k*_O_2__ as the rate-determining step which does not involve the CAN oxidant. Berlinguette and coworkers found that introduction of electron-donating methoxy groups at the 4,4′ positions of the bpy ligands enhances the catalytic efficiency of **1a**.^44^ For the modified complex (**1b**), a TOF of 1.5 × 10^–4^ s^–1^ was obtained under the same conditions as were applied for **1a**, and the rate constants *k*_e_ and *k*_O–O_ were measured as 3.7 M^–1^ s^–1^ and 3.0 × 10^–5^ s^–1^ (Table 1) respectively by using stopped-flow techniques.^44^ Unlike **1a**, the rate of CAN consumption for **1b** is first order relative to both the catalyst and CAN with a rate constant smaller than *k*_e_. Therefore, the oxidation of [Ru^IV^–OO] to [Ru^V^–OO] was proposed to be the rate-limiting step in the catalytic cycle of **1b**. Yagi *et al.* reported that electron-donating groups on the tpy moiety of **1a** also remarkably improve the catalytic performance.^49^ Llobet and coworkers found that the fluoride substituents at the 6,6′ positions of bpy ligand of **1a** not only perturb the electronic feature but also act as internal base.^50^ Complexes **2a–2c** are composed of the same polypyridine ligands as **1a** but with halogens instead of the aquo ligand in **1a**. They show catalytic water oxidation activity in aqueous medium. It is believed that they convert to **1a** by dissociation of the halogen ligand in the aqueous environment and the resulting aqua complex **1a** plays the role of an authentic catalyst.^45,46^ In the model complex [Ru^II^(tpy)(pynp)OH_2_]^2+^ (**3b**, pynp = 2-(pyrid-2′-yl)-1,8-naphthyridine), the bpy ligand is annulated with another pyridyl moiety which does not ligate with the Ru center but is hypothesized to act as an internal basic site.^51,52^ The opposite orientation of the asymmetric pynp ligand leads to two geometric isomers for **3b** that show a significant difference in electrochemical properties and catalytic performance for water oxidation. The TOF (4.8 × 10^–4^ s^–1^) of the *cis*-isomer, in which the uncoordinated naphthyridine nitrogen atom is in the vicinity of the aqua ligand, is much less than the TOF (3.8 × 10^–3^ s^–1^) of *trans*-isomer under the conditions of 500 equivalents CAN and pH = 1.0.^52^ How the uncoordinated nitrogen site might regulate the catalytic activity is not yet clear.

**Table 1:** Rate constants of selected Type I WOCs following the general catalytic cycle of Scheme 5

WOC	*k*_e_ (M^–1^ s^–1^)	*k*_O–O_ (s^–1^)	*k*_O_2__ (s^–1^)	TOF^*a*^ (s^–1^)
**1b** ^44^	3.7	3.0 × 10^–5^	—^*b*^	1.5 × 10^–4^
**3a** ^38^	5.0	9.6 × 10^–3^	7.4 × 10^–4^	1.9 × 10^–4 ^ ^*c*^
**5** ^42^	1.7 × 10^3^	1.1 × 10^–2^	—^*b*^	1.7 × 10^–1^

^*a*^Large excess of CAN in 0.1 M HNO_3_.

^*b*^Not available.

^*c*^According to the decay of CAN (30 equivalents).

Compared to the bpy ligand in **1a**, the 2,2′-bipyrimidine (bpm) ligand of **3a** elevates the redox potential of the Ru^III/II^ couple and reduces the potential of the Ru^IV/III^ couple to such an extent that the former is more positive than the latter.^38,41^ As a result, [Ru^II^(tpy)(bpm)(OH_2_)]^2+^ (**3a**) undergoes a proton-coupled two-electron [Ru^IV^ =O]/[Ru^II^–OH_2_] event in the pH range 0–9.7 (p*K*_a1_ of [Ru^II^–OH_2_]) as illustrated in the Pourbaix diagram (red line in Fig. 3). Furthermore, a [Ru^V^ =O]/[Ru^IV^ =O] redox wave at 1.65 V was observed in the cyclic voltammogram of **3a**. A kinetic study suggested a rate constant *k*_O–O_ = 9.6 × 10^–3^ s^–1^ for the O–O bond forming step, which is considerably greater than that for **1b** (Table 1). This observation implies much stronger electrophilicity of the [Ru^V^ =O] intermediate derived from **3a** than that derived from **1b**. An outcome from the rapidity of O–O bond formation is that O_2_ liberation from [Ru^IV^(OO)]^2+^ (*k*_O_2__ = 7.4 × 10^–4^ s^–1^) becomes the slowest and rate-determining step in the catalytic cycle of **3a**.

Besides various bidentate ligands,^53^ several tridentate ligands in place of tpy have been incorporated into complexes of the [Ru(LLL)(LL)OH_2_] motif, which are able to catalyze water oxidation. WOC **4**, for example, possesses a 2,6-bis(1-methylbenzimidazol-2′-yl)pyridine (Mebimpy) ligand that has a stronger σ-donating ability than tpy.^40,41^ Unlike **1a**, complex **4** tentatively undergoes the oxidation of either the [Ru^IV^ =O] or [Ru^IV^–OO] intermediate as the rate-determining step. Complex **5** contains a negatively charged 2,2′-bipyridine-6-carboxylate (bpc) ligand that can donate lone pair electrons of oxygen to stabilize the high-valent Ru center *via* pπ–dπ interaction.^42^ The advantage of introducing the anionic carboxylate donor can be understood by comparison of **1a**, **3a** and **5** (see Fig. 3 and Table 1). For **5** we observed a slight decrease in the potential (1.57 V) but a dramatic increase in the kinetics (*k*_e_ = 1.7 × 10^3^) of the [Ru^V^ =O]/[Ru^IV^ =O] electron transfer step. Although the rate constant *k*_O_2__ for **5** can not be probed experimentally, it is assumed to be greater than the rate constant (*k*_O–O_ = 1.1 × 10^–2^ s^–1^) of the O–O bond formation step claimed as rate-limiting in the catalytic cycle of **5**, and thus significantly greater than *k*_O_2__ (7.4 × 10^–4^ s^–1^) for **3a**. Apparently the carboxylate group facilitates dioxygen release from the Ru center. A TOF of 1.7 × 10^–1^ s^–1^ identifies complex **5** as the fastest WOC exhibited in the Type I group of selected candidates. The tertiary amine groups of tridentate dmap (2,6-bis(dimethylamino)pyridine) ligand are stronger σ-donor than pyridine of tpy. As a result, the Ru^III/II^ and Ru^IV/III^ redox potentials of [Ru^II^(dmap)(bpy)(OH_2_)]^2+^ are less positive than those of **1a** under neutral conditions. A recent study reveals that [Ru^II^(dmap)(bpy)(OH_2_)]^2+^ is capable of catalyzing water oxidation with a slow rate *via* a rate-determining O–O bond formation step (*k*_O–O_ = 2.0 × 10^–2^ s^–1^).^54^

### 3.4 Type II: [Ru(LLL)(L)_2_X]^*n*+^ WOCs

**Fig. 4:**
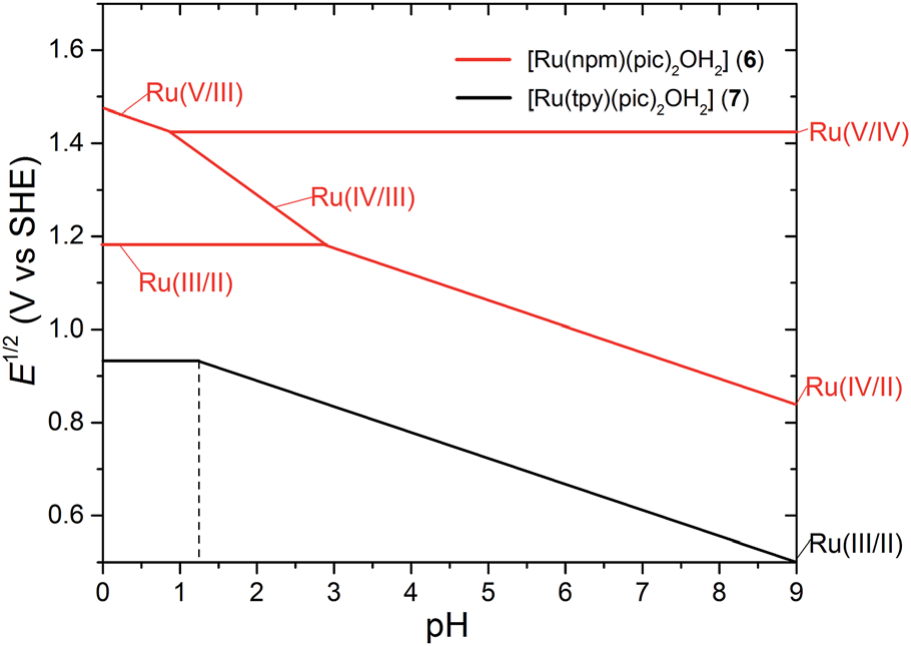
Pourbaix diagrams for [Ru(npm)(pic)_2_OH_2_]^2+^ (**6**) and [Ru(tpy)(pic)_2_OH_2_]^2+^ (**7**); solid lines indicate trends of redox potentials depending on pH; dotted lines indicate p*K*_a_ of [Ru^III^–OH_2_] species. The diagram was drawn according to reported experimental data in refs. 55 and 57.

**Scheme 6:**
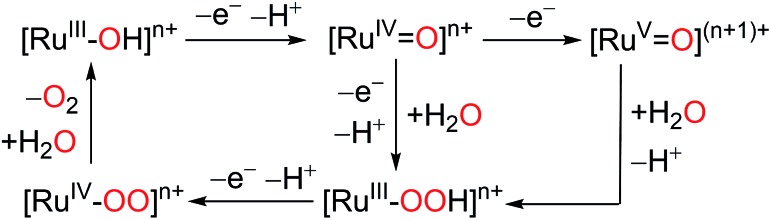
Proposed mechanism for water oxidation by complex **6** in aqueous medium.

Unlike the Type I WOCs discussed in the previous section, Type II complexes bind three monodentate ligands in addition to a tridentate ligand. The aqua ligand, if there is one, always occupies the fourth binding site in the equatorial plane defined by the ruthenium and the tridentate ligand. One of the earliest examples of this group is [Ru^II^(npm)(pic)_2_OH_2_]^2+^ (**6**, npm = 4-*t*-butyl-2,6-di-(1′,8′-naphthyrid-2′-yl)-pyridine) prepared by Thummel and coworkers in 2005.^33^ The single-crystal X-ray structure of **6** shows that the two external 1,8-naphthyridyl nitrogens do not coordinate with Ru^II^ but one of them does form an H-bond with the coordinated water. The higher p*K*_a1_ of **6** (>13.5) as compared to **7** (11.2) indicates that the intramolecular H-bond inhibits proton dissociation from the bound water. The pH dependence of the redox potentials of **6** in aqueous solution is summarized in a recent mechanistic study.^55^ The pH slope of –59 mV pH^–1^ in the pH > 2.9 region of the Pourbaix diagram (Fig. 4) is attributed to a two-proton coupled two-electron [Ru^IV^ =O]/[Ru^II^–OH_2_] oxidation. The situation is different in the lower pH region. The independence of the redox potential relative to pH suggests a [Ru^III^–OH_2_]/[Ru^II^–OH_2_] process. Further [Ru^V^ =O]/[Ru^IV^ =O] oxidation occurs at 1.42 V over a wide pH range from 0.9 to 10. A very significant finding in this study is the identification of a [Ru^IV^–OO]^2+^ species, the formation of which requires even lower thermal energy than the formation of the [Ru^V^ =O] intermediate. Based on combination of experimental and theoretical results, the authors proposed a catalytic cycle (Scheme 6) for **6** that involves two competing O–O bond formation pathways. The generation of the [Ru^III^–OOH]^2+^ intermediate can proceed *via* either water nucleophilic attack on a [Ru^V^–O]^3+^ species or the net reaction between [Ru^IV^–O]^2+^ and a water molecule accompanied by the loss of an electron and a proton. While a DFT simulation predicted a similar thermodynamic energy change for these two pathways under standard conditions (pH = 0), the latter pathway is more favored at higher pH since it is a proton-coupled process and the former one is not.^56^

Complex **7** has a coordination geometry very similar to **6**. However, it does not possess any vacant nitrogen site that can form an H-bond with a bound water. The electrochemical behavior of **7** as displayed in the Pourbaix diagram (Fig. 4) is quite different from that of **6**.^57^ PCET couples corresponding to [Ru^III^–OH]/[Ru^II^–OH_2_] appeared in a broad region. The p*K*_a1_ values of [Ru^III^–OH_2_] and [Ru^II^–OH_2_] were deduced from the potential/pH relationship as 1.2 and 11.2, respectively. Under acidic and neutral conditions, a prominent catalytic current was observed in the cyclic voltammogram of **7** with an onset that was clearly separated from the Ru^III^/Ru^II^ redox wave. While it is determined that a ruthenium species of higher oxidation state than Ru^III^ is needed to trigger water oxidation, no redox wave can be distinguished unambiguously for further oxidation of the Ru^III^ intermediate at pH < 10. Thus one can speculate that the [Ru^IV^ =O] form of **7** is responsible for O–O bond formation in the same fashion as **6**. The mechanistic details of **7** have not been elucidated, however, the TOF (1.3–3.7 × 10^–2^ s^–1^)^57,58^ of **7** is very close to the TOF (3.2 × 10^–4^ s^–1^)^56^ of **6**, measured in CAN-driven O_2_ evolution experiments.

The catalytic activity has been investigated for complexes **8a–8c**, in which halogen ligands instead of aqua occupy the equatorial coordination site.^58^ A 10–12 min induction period (the concentration of catalyst is 0.04 mM) was observed before **8a** and **8b** began to catalyze O_2_ evolution in the presence of excess CAN. Their TOFs are lower than that of [Ru^II^(tpy)(pic)_2_OH_2_]^2+^ (**7**). These observations are consistent with the suggestion that halogen/water exchange is required to generate the authentic WOC **7**. On the contrary, the iodide complex [Ru^II^(tpy)(pic)_2_I]^+^ (**8c**) catalyzed CAN-driven O_2_ evolution without any induction period and achieved a TOF of 0.16 s^–1^ that is greater than its aqua analog **7**. This unusual catalytic performance of **8c** suggests a mechanism that involves the iodide group and differs from what is proposed for **6** or **7**. Thus far no insights regarding this concern have been revealed.

Complexes **9** and **10** preserve the same coordination geometry as other Type II complexes.^47^ Otherwise, there is no aqua or ‘labile’ halogen monodentate ligand. By comparing **9** and **10**, it is found that a dianionic carboxylate ligand, rather than the neutral tpy, facilitates picoline/water exchange at the Ru^III^ state.^59^ DFT model studies estimate a lower energy barrier for **10** than **9** by about 10 kcal mol^–1^, corresponding to a remarkably faster picoline/water exchange rate for **10**. This accelerated exchange rate is attributed to destabilization of the ruthenium d_*z* ^2^_ orbital by carboxylate, resulting in a large energy gap between the binding orbitals of Ru and picoline. We suggest that the aqua complex [Ru^III^(pdc)(pic)_2_OH_2_] (pdc = 2,6-pyridine-dicarboxylate) derived from **10** is the actual WOC initiating catalytic O_2_ evolution. The TOF (0.23 s^–1^) of **10** is significantly greater than that of **7**. The introduction of an amide group in place of one carboxylate group of pdc further lowers the oxidation potential and enhances the catalytic activity of the complex.^60^

### 3.5 Type III: [Ru(LLLL)(L)_2_]^*n*+^ WOCs

**Scheme 7:**
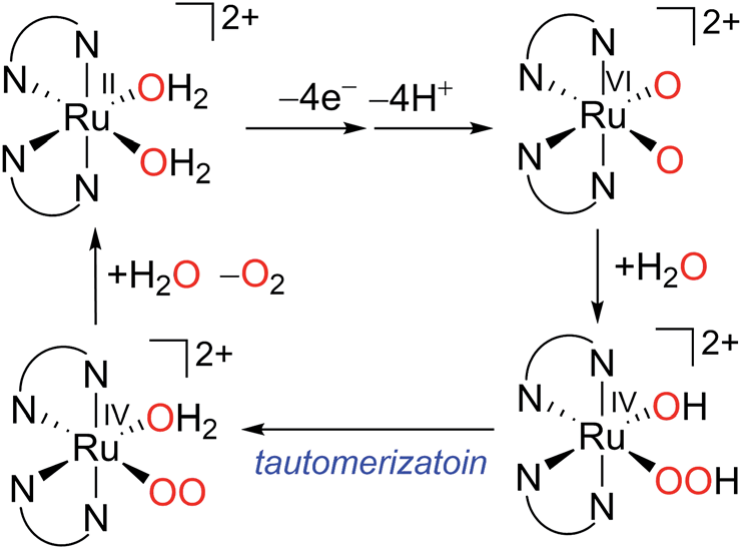
Proposed mechanism for water oxidation by complex **11** in aqueous medium.

The complex *cis*-[Ru^II^(bpy)_2_(OH_2_)_2_]^2+^ (**11**) has the same coordination environment as either ruthenium site of the blue dimer where an aqua ligand replaces the oxo-bridge. Thus **11** represents a monomeric analog of the blue dimer. An electrochemical study by Meyer *et al.* showed that complex **11** can lose 4H^+^/4e^–^ in a stepwise fashion within a narrow potential range 0.8–1.5 V *vs.* NHE and form a Ru bis–oxo complex *cis*-[Ru(bpy)_2_(O)_2_]^2+^ with a formally VI ruthenium center.^61^ Mixing **11** and CAN in 0.1 M CF_3_SO_3_H resulted in O_2_ evolution and RuO_2_ precipitation simultaneously.^61,62^ Therefore, there is some uncertainty about whether **11** or RuO_2_ actually catalyzes water oxidation. In a more recent study, Llobet and coworkers revisited complex **11**.^63^ They found that *cis*-[Ru^II^(bpy)_2_(OH_2_)_2_]^2+^ is capable of catalyzing dioxygen production at a much faster rate than either its *trans*-isomer or RuO_2_, although the catalytic performance of **11** is limited to several turnovers. An ^18^O-labeling experiment demonstrated that the dioxygen evolved from the first catalytic cycle originated from both the complex aqua ligand and the solvent water molecules. This result supports an ‘acid–base’ pathway, as depicted in Scheme 7, and rules out intramolecular O–O bond formation. A DFT simulation computed the activation free energy of the water nucleophilic attack and O_2_ release steps to be 24.5 and 25.1 kcal mol^–1^, respectively. Both values are greater than the activation energy of the tautomerization step.

**Fig. 5:**
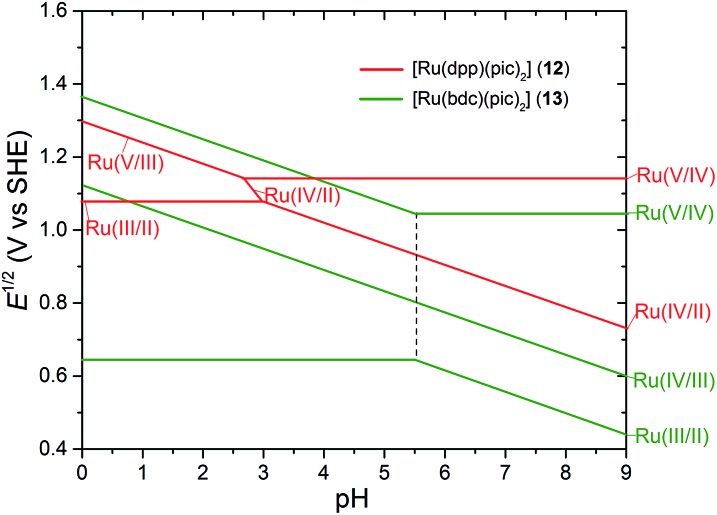
Pourbaix diagrams for [Ru(dpp)(pic)_2_]^2+^ (**12**) and [Ru(bdc)(pic)_2_] (**13**); solid lines indicate trends of redox potentials depending on pH; dotted lines indicate p*K*_a_ of [Ru^III^–OH_2_] species. The diagram was drawn according to reported experimental data in ref. 64 and 65.

**Scheme 8:**
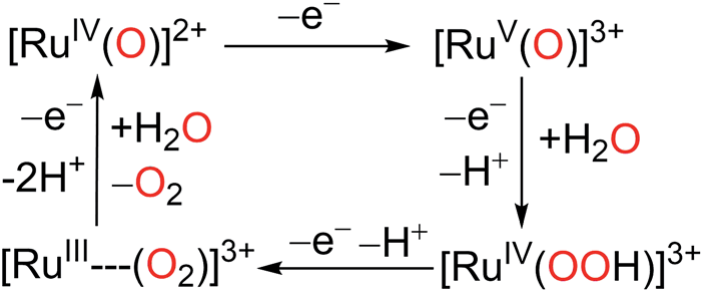
Proposed mechanism for water oxidation by complex **12** in aqueous medium.

Complex **11** is prone to lose bpy ligands when it is oxidized to a high oxidation state.^61^ This loss is attributed to the rapid decomposition of **11** under water oxidation conditions and thus the low catalytic turnover. The *trans*-isomer of **11**, however, is more stable with respect to ligand dissociation. Thummel and coworkers incorporated a rigid phenanthroline moiety to replace the central bpy of qpy (2,2′:6′,2′′:6′′,2′′′-quaterpyridine) thus preparing the tetradentate ligand 2,9-di-(pyrid-2′-yl)-1,10-phenanthroline (dpp), in which rotation about the central bpy–bpy bond has been restricted.^66,67^ Complex **12** involving the equatorial tetradentate dpp ligand and two axial pic ligands is the earliest example in the category of [Ru(LLLL)(L)_2_] WOCs.^47,67^ In the presence of CAN, **12** was reported to catalyze O_2_ evolution with a TOF of 1.2 × 10^–2^ s^–1^.^68^ Although no water is coordinated with the Ru(ii) center of **12**, its Pourbaix diagram (Fig. 5) clearly demonstrates features of PCET processes. Theoretical studies corroborated that, in the medium and high pH regions, complex **12** accommodates a water molecule during the 2e^–^/2H^+^ PCET oxidation resulting in a seven-coordinate 18-electron [Ru^IV^(O)]^2+^ intermediate. A consequent redox event at 1.14 V was assigned to the [Ru^V^(O)]^3+^/[Ru^IV^(O)]^2+^ process. In the low pH region, on the other hand, the pathway involves [Ru^III^]^3+^/[Ru^II^]^2+^ ET and follows 2e^–^/2H^+^ PCET redox steps to produce a seven-coordinate [Ru^V^(O)]^3+^ species. Water association to the ruthenium center is presumed to occur concurrent with the redox process. DFT simulation proposes an ‘acid–base’ mechanism for the O–O bond formation between seven-coordinate [Ru^V^(O)]^3+^ intermediate and water.^64^ It requires a calculated thermodynamic potential of 1.94 V that is the highest in the predicted catalytic cycle (Scheme 8) for **12**. It should be noted that the X-ray structure of **12** shows a considerably large 125° external N–Ru–N (dpp) angle. This feature might facilitate water insertion in the primary coordination sphere of the complex. A recent study on several analogs of **12** indicates that both electronic and steric modification affects the catalytic performance.^68^ It is difficult, however, to establish a straightforward structure–activity correlation.

The Pourbaix diagram (Fig. 5) of complex **13** shows quite different features from that of **12**.^65^ Firstly, the redox potentials of Ru^III/II^, Ru^IV/III^, and Ru^V/IV^ are well separated over the whole pH range from 0 to 12. Secondly, the Ru^III^/Ru^II^ oxidation process is coupled with proton transfer when the pH is higher than 5.5, indicating water molecule association in the redox step because complex **13** in its divalent state does not bind an aqua ligand. Thirdly, the IV oxidation state of **13** can be reached at a lower potential than that of **12**. At pH = 1.0, for example, the Ru^IV/III^ redox potential is about +1.05 V. Sun's group successfully isolated the Ru^IV^ species from pH = 1.0 aqueous solution as a dimeric {μ-(HOHOH)[Ru^IV^(bdc)(pic)_2_]_2_}^3+^ (bdc = 2,2′-bipyridine-6,6′-dicarboxylate) complex, of which each Ru^IV^ center is seven-coordinated incorporating one hydroxyl ligand in the equatorial plane.^69^ The structure of the Ru^IV^ species might be stabilized by a hydrogen bonding network including a solvated water molecule, hydroxyl ligand, and carboxylate groups. It implies a possible proton-shuttling path from the hydroxy ligand to the bulk solvent during water oxidation.

**Scheme 9:**
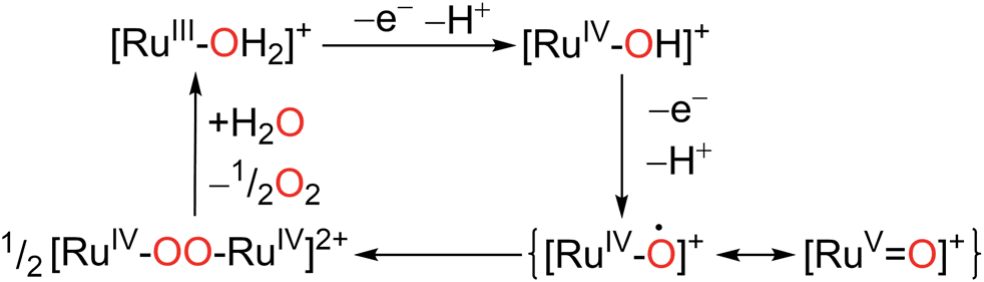
Proposed mechanism for water oxidation by complex **13** in aqueous medium.

A cyclic voltammogram of **13** under acidic conditions showed the onset of a catalytic current at a more positive potential than the Ru^V^/Ru^IV^ redox potential. A kinetic study at pH = 1.0 using a stopped-flow technique suggests a catalytic cycle for **13** as displayed in Scheme 9.^65^ The O–O bond was proposed to form *via* coupling of two [Ru^V^ =O]^+^ species which can be regarded as a resonance form of the Ru^IV^ oxyl radical [Ru^IV^–O˙]^+^. Dioxygen release from the resulting [Ru^IV^–OO–Ru^IV^]^2+^ intermediate was believed to be the rate-determining step under stoichiometric CAN conditions. In the presence of excess CAN, however, [Ru^IV^–OO–Ru^IV^]^2+^ can be rapidly oxidized to a superoxo [Ru^IV^–O˙O–Ru^IV^]^3+^ intermediate which liberates O_2_ at a fast rate. As a result, the radical coupling step becomes rate-determining. This hypothesis is supported by the experimental observation that the rate of water oxidation by **13** was second order with respect to the catalyst when a large excess of CAN was used.^65^ The complex was reported to be capable of catalyzing water oxidation with a TOF of 12 s^–1^. Electron withdrawing and hydrophobic substituent groups on the axial ligands boost the catalytic activity.^70^ In one case, where isoquinoline was introduced as the axial ligand, an astonishing TOF of 303 s^–1^ was obtained.^65^ This elevated rate is attributed to the noncovalent intermolecular attraction between isoquinolines which lowers the energy barrier for the radical coupling step. The systematic study of Ru WOCs with bdc ligands, including **13** and its analogues, has recently been reviewed by Sun *et al.* ^71^

Concepcion and coworkers prepared the complex [Ru^II^(bdp)(pic)_2_] (H_2_-bdp = 2,2′-bipyridine-6,6′-diphosphonic acid) as a phosphonate analog of **13**.^12^ Using CAN as an oxidant at pH = 1.0, [Ru^II^(bdp)(pic)_2_] is found to catalyze water oxidation *via* an acid–base pathway involving a seven-coordinate [Ru^IV^–OH]^–^ intermediate and a rate-limiting oxidation step. The TOF (0.3 s^–1^, assuming 100% CAN efficiency) of [Ru^II^(bdp)(pic)_2_], however, is almost two orders of magnitude smaller than the TOF of **13**, highlighting the favoured radical coupling rather than acid–base mechanism for a highly efficient catalyst. Llobet *et al.* investigated the catalytic water oxidation behavior of [Ru^II^(tda)(py)_2_] (H_2_-tda = 2,2′:6′,2′′-terpyridine]-6,6′′-dicarboxylic acid), in which the pentadentate tda ligand contains one pyridine moiety more than bdc.^24^ The authors proposed a seven-coordinate Ru^V^ =O state of the complex with a dangling carboxylate group that can form H-bond with incoming water molecule and thus facilitate the electrophilic attack of the oxo to the water molecule. An impressive TOF of 8000 s^–1^ at pH 7.0, assessed by electrochemical method, makes [Ru^II^(tda)(py)_2_] the most efficient mononuclear WOC ever reported. Chemical-driven water oxidation catalysis for the complex was not revealed in the study.

### 3.6 Type IV: [Ru(LLL)_2_]^*n*+^ WOCs

The primary coordination sphere of [Ru^II^(tpy)_2_]^2+^ is saturated by six rigid Ru–N(tpy) coordination bonds. The complex does not possess a vacant coordination site to accommodate a water molecule and replacement of one of the bound pyridines by water has never been observed. To behave as a WOC therefore, [Ru^II^(tpy)_2_]^2+^ must expand its coordination sphere to seven by the addition of a water molecule, much like complex **12**. Such hepta-coordination demands a pentagonal bipyramid geometry which would dictate the impossible situation of a single tpy ligand spanning both axial sites with the Ru-tpy coordination (N–Ru–N) arranged in an approximate linear fashion. When the tpy ligand binds with a single metal center it forms two adjacent five-membered chelate rings that define an exterior N–Ru–N angle of only about 158°. If the size of one of these chelate rings is increased from five to six, however, the resulting ligand could span both axial sites. The tridentate ligand 2-(quinol-8′-yl)-1,10-phenanthroline (phenq) binds Ru(ii) as a 6-5 chelator and thus can accommodate 7-coordinate pentagonal bipyramid geometry. The [Ru(phenq)(tpy)]^2+^ complex (**15**) thus shows modest WOC activity (TON = 334).^72^ Several other Ru(ii) complexes involving tridentate 6-5 chelators have likewise been shown to be active as WOCs, pointing to the importance of conformational effects in designing active catalyst systems. It is possible that the ruthenium center coordinates with a water molecule at high valent state, such as Ru(iv), which is more electron-deficient than the divalent state. Meanwhile, the complex has to reorganize its structure to provide space in the coordination sphere for the association of an oxygen (water) ligand. This reorganization might be accomplished by weakening and elongation of certain N–Ru coordination bond. We expect that the substitution of quinoline for pyridine in some of the ligand systems shown in Scheme 1 will provide an interesting and useful new family of metal binders.

### 3.7 Auxiliary pathways contribute to dioxygen evolution

Chemical-promoted catalytic water oxidation is usually performed in the presence of a large excess of a sacrificial oxidant, hundreds to thousands of equivalents relative to the amount of the Ru catalyst. Partly due to such harsh conditions competing pathways have been observed and proposed to contribute to O_2_ evolution concomitant with the primary catalytic pathways described above. Berlinguette and co-workers found that not all oxygen atoms of dioxygen were derived from water when they studied water oxidation catalyzed by **1a**.^44^ They proposed intermolecular oxygen atom abstraction from NO_3_^–^ by a high-valent [Ru=O] species under mediation of the Ce^IV^ cation. This result is corroborated by the detection of NO_2_ in the catalytic reaction system. Moreover, MS/MS techniques have trapped a dioxygen [Ru^III^–OO]^+^ fragment, as the product of oxygen atom transfer, from the MS signal corresponding to the {[Ru(tpy)(bpy)O][Ce(NO_3_)_5_]}^+^ cluster ion.^44^

**Scheme 10:**
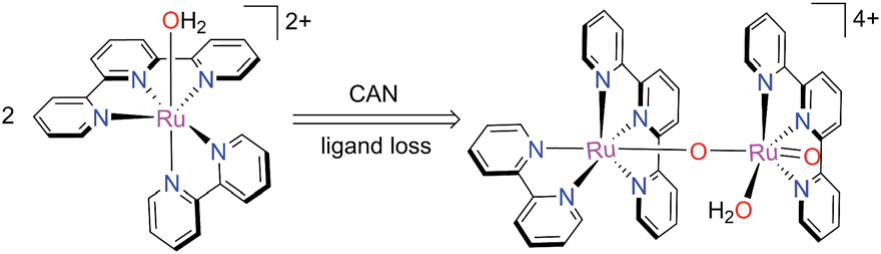
Generation of oxo-bridged dinuclear catalyst during the CAN-promoted catalytic water oxidation process.

Llobet and co-workers reported that the mononuclear catalyst **1** could lose its bpy ligand and convert to an oxo-bridged dinuclear [Ru^IV^–O–Ru^IV^ =O]^4+^ species (Scheme 10) *in situ* during CAN-promoted water oxidation.^73,74^ They managed to isolate the dinuclear complex and characterized its structure by single-crystal X-ray diffraction. This conversion is slow but irreversible through a self-assembly type process. While the dinuclear complex exhibited catalytic activity similar to **1** towards water oxidation, it is a more robust WOC than **1**. A DFT calculation supported a catalytic cycle for the dinuclear species which coexisted in parallel with the catalytic cycle of the mononuclear catalyst **1**. Very recently, Sakai and co-workers found that catalyst **13** could lose monodentate pyridine ligands and assembled to a trimeric ruthenium species upon oxidation in a very similar manner as **1**.^75^ The isolated trinuclear ruthenium complex has a ‘Ru^III^–O–Ru^IV^–O–Ru^III^’ motif with μ-oxo-bridges. In a light-driven [Ru(bpy)_3_]^2+^/S_2_O_8_^2–^ photochemical system (pH = 8.0), it is capable of catalysing O_2_ evolution with a TOF of about 0.9 s^–1^. Mechanistic details of the trinuclear Ru complex are under investigation.

**Scheme 11:**
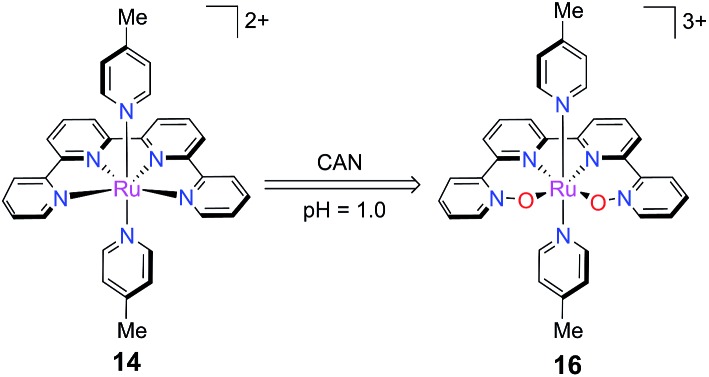
Oxidation of polypyridine ligand into *N*-oxide ligand during the CAN-promoted catalytic water oxidation process.

Investigation of complex **14** by Lau and co-workers revealed that the qpy ligand was oxidized to qpy-*N*,*N*′′′-dioxide in a pH = 1.0 aqueous solution of CAN (Scheme 11).^76^ The resulting Ru(iii) complex was isolated and structurally characterized by single-crystal X-ray diffraction. It showed a considerably shorter induction period (about 1 min) as compared to **14** (about 5 min) in CAN-promoted O_2_ evolution experiments. After the induction period, the O_2_ evolution rates for **14** and its di-*N*-oxide counterpart are comparable. ^18^O-Labeling experiments indicated that the oxygen atoms of the di-*N*-oxide are not found in the catalytically generated dioxygen. These observations imply oxidative conversion of the qpy ligand as part of the Ru complex with the di-*N*-oxide **16** being the authentic catalyst for water oxidation. The kinetics of qpy to qpy-*N*,*N*′′′-dioxide, however, have not been disclosed in detail. It is not clear yet if there are competing catalytic pathways that might include both **14** and **16**.

## 4. Conclusions and outlook

A series of 15 mononuclear Ru(ii) polypyridine complexes have been selected as representative WOCs to review and divided into four groups according to the disposition of the pyridine ligands around the metal center. Type I complexes have a tridentate, bidentate, and monodentate ligand occupying the 6 coordination sites of Ru(ii). Type II have a tridentate and three monodentates. Type III are (mostly) tetradentate in the equatorial plane plus two axial monodentates. Finally, Type IV is unique with two tridentate ligands binding in a meridional fashion. While all multi-dentate ligands are polypyridine based, the monodentate ligand could be a water, halogen, or a substituted pyridine.

The aqua ligand can release protons upon oxidation of the Ru(ii) complex. Such a PCET process is essential to the formation of a high valent, Ru(iv) or Ru(v), ruthenium oxo species at a moderate potential. There are Ru(ii) examples in every group that do not possess an aqua ligand. They coordinate with a water molecule by ligand exchange or reorganization of structure to provide a vacant binding space.

The catalytic activity of the Ru(ii) complexes is evaluated by analysis of the profiles of oxygen evolution *vs.* time. Since only the oxidative half of the water splitting reaction is under scrutiny, a sacrificial oxidant must be used in a stoichiometric fashion. Typically we have used ceric ammonium nitrate under acidic conditions as this sacrificial reagent. The catalytic activity of these Ru(ii) complexes towards water oxidation has been discussed in light of two fundamentally different mechanisms: one involving attack of a water molecule on the oxygen of a high valent Ru=O species and the other involving the formation of a Ru–O˙ radical that could then dimerize to give a Ru–O–O–Ru species. The former mechanism appears to be the most prevalent for the systems under discussion. Only complex **13**, among all the candidates, prefers the latter mechanism. Meanwhile **13** is the most active WOC in terms of TOF in CAN-driven O_2_ evolution experiments. Its superior activity evokes a putative favoring of the radical coupling pathway for a highly efficient WOC. It appears that the fundamental differences in mechanism among the four types of complexes involve the chemistry of the critical higher valent ruthenium oxo intermediates. On one hand, the Ru=O intermediates trigger O–O formation; on the other hand, they represent the highest formal valence of the ruthenium center in the catalytic cycle.

Isolation of high valent Ru=O intermediates *in situ* is quite challenging due to their thermal instability and the strong solvation effect of the prerequisite aqueous medium. The structures of the Ru=O intermediates are related to the arrangement of the coordinating ligands. It is reasonable to envision the location of oxo ligand outside and inside the plane of tridentate polypyridine ligand for Type I and II WOCs, respectively. For Type III WOCs, a seven-coordinate structure seems favored for the Ru oxo intermediate. Transient spectroscopic techniques are able to probe kinetics in the catalytic cycle. Thus far, O–O bond formation, electron transfer, or O_2_ liberation have been proposed as rate-determining for different WOCs.

The complicated mechanistic details, especially the various rate-limiting steps, of diverse WOCs make it almost impossible to establish a universal correlation between the structure and activity of WOCs. Nevertheless, there are some basic principles that can be applied to individual steps regardless of catalyst group. For example, the anionic carboxylate ligand has been found to enhance the rate of O_2_ liberation step for both Type I and Type II WOCs. The introduction of electron-donating substituents, in general, facilitates the electron transfer process.

The design of homogeneous transition metal WOCs should meet some basic requirements: access to the metal–aqua and metal=O states, validity of O–O bond formation, and stability and solubility in aqueous solution. It is important to target the rate-determining step of the catalytic cycle. The goal is to reduce the activation energy of this step by modification of the ligands. Specific ligand features can be considered involving both the inner and outer coordination spheres. The former includes ligand properties such as rigidity, conjugation, σ/π-donating ability, coordination vacancy, and interaction of ligand donors. The latter includes the electronic effect of substituents, hydrogen bonding properties, steric repulsion and hydrophilicity.

Looking to the future, there is a mounting effort to extend redox catalysis to include more earth abundant metals, especially the first row transition metals. Both Co and Ni have been widely investigated as proton reduction catalysts to produce hydrogen and Fe, Co, Mn, and others have been used in systems active towards water oxidation. As with Ru(ii), it is the ligand environment that will ultimately control the redox activity. Lessons learned from ruthenium-based catalysts should inspire and motivate the development of catalysts based on other transition metals. The future promises considerable new development directed towards the realization of a practical system for artificial photosynthesis.
